# Comment on the case for considering quality of life in addiction research and clinical practice

**DOI:** 10.1186/1940-0640-7-2

**Published:** 2012-03-15

**Authors:** Robert G Newman

**Affiliations:** 1 International Center for Advancement of Addiction Treatment, Baron Edmond de Rothschild Chemical Dependency Institute, Beth Israel Medical Center, New York, NY, USA

## 

In making the case for considering quality-of-life outcomes in the treatment of addiction (Addiction Science & Clinical Practice, July 2011 [1]), Dr. Alexandre Laudet argues that "reduced substance abuse is not in itself an adequate criterion for recovery [p. 49]." In this respect, however, management of drug dependence is no different than the almost universal approach to assessing care of all other chronic medical conditions. For example, in treating patients with diabetes or hypertension, the overwhelming (usually exclusive) focus is on the maintenance of blood sugar and blood pressure levels; in the treatment of epilepsy, efficacy is a function of the elimination or reduction in frequency of seizures. It is difficult to argue with Dr. Laudet's view that it would be appropriate and useful to ask substance abuse patients, "Overall, how satisfied are you with your life?", but how often is this question posed by clinicians treating other chronic medical ailments?

Paradoxically, providers of substance abuse treatment demonstrate both too much and too little focus on quality-of-life parameters. Thus, with regard to medication-assisted treatment, most programs set criteria for "termination" based on attendance, adherence to counseling schedules, and urine toxicology results, and they apply these criteria rigidly, with no regard whatsoever as to how favorably the patient judges her or his improved overall condition. Other programs, however, make demands on patients that far transcend the primary problem they treat--drug dependence--and in some cases even discharge patients who fail to find employment within a specified period of time, even when illicit drug use has ceased altogether [2].

The best approach would seem to be to apply to addiction treatment precisely the same perspectives and practices, good and bad, as are commonplace in the care of all other chronic ailments.

## Author Reply

Dr. Newman raises interesting points that are frequently mentioned when quality of life is discussed in the context of substance use disorders (SUDs). Thank you for the opportunity to offer some clarifications.

Pointing out that the nearly exclusive focus of treatment for other chronic conditions-- e.g., asthma--is on reducing and managing symptoms, Dr. Newman writes, "The best approach would seem to be to apply to addiction treatment precisely the same perspectives and practices, good and bad, as are commonplace in the care of all other chronic ailments." From the perspective of specialty care (i.e., addiction treatment), especially medication-assisted treatment, it may seem difficult to disagree: Clients are diagnosed with a physical condition and prescribed the most effective pharmacotherapy for their diagnosis.^a^

However, this loses sight of the fact that SUDs are somewhat unique among chronic conditions in that, for many, they typically present with numerous co-occurring functional impairments (physical- and mental-health problems, of course, but also housing instability, damaged family and social relations, and poor job readiness or employability). These domains are what constitute "quality of life." As argued in my article, improvement in these impaired areas of functioning are desirable and, some would argue, necessary, for reductions in or cessation of substance misuse to be optimized and sustained. The suggestion that we ask patients, "Overall, how satisfied are you with your life?" clearly requires scientific investigation to determine the most effective way to obtain a preliminary sense of a patient's overall quality of functioning. Note that, in the primary-care setting, although doctors may not inquire about quality of life *per se*, they often ask about stress level and/or major changes or difficulties in the patient's life that might better inform care.

In closing, some misconceptions that often fuel objections to considering quality of life in the context of SUD treatment should be addressed. First, the argument implies that clinicians be held accountable for making clients "happy." This is a gross oversimplification. Clinicians in any field are accountable, first and foremost, for facilitating symptom reduction. However, because of the high prevalence of co-occurring functional impairments among SUD patients, and of the emerging evidence that functional improvements prospectively enhance motivation for, and the likelihood of, sustained abstinence, providing SUD-affected persons with the tools and resources to improve functioning is highly desirable. In the current service-delivery system, this may take the form of referrals to vocational or housing services when such services are not integrated in the treating agency.

Second, as discussed in the article, the full relevance of considering quality of life in the treatment of addiction is in the context of recovery-oriented systems of care (ROSC), whose central goal is to promote not just symptom reduction but recovery (defined as abstinence and improvements in functioning). The intensive, time-limited specialty-care treatment model is well-suited to initiate remission (symptom reduction), but it is not sufficient to, nor was it designed to, promote recovery as currently conceptualized. Recovery is a multifaceted process that unfolds over time and goes well beyond symptom reduction. For many, it requires far more than a brief episode of professionally delivered SUD treatment, as evidenced by the increasing body of science supporting the usefulness of post-treatment services and of adopting a recovery orientation to address SUD.

Alexandre B. Laudet, PhD

Center for the Study of Addictions and Recovery, National Development and Research Institutes, Inc.,

New York, NY, USA.

Email: Alexandre B. Laudet - laudet@ndri.org

## Endnote

^a^Editor's note: Quality of life is relevant to pharmacotherapies in that the Food and Drug Administration requires evidence of patient reported outcomes, including quality of life, to support labeling claims [3].
